# Incidence and predictors of readmission within 30 days of transurethral resection of the prostate: a single center European experience

**DOI:** 10.1038/s41598-018-25069-5

**Published:** 2018-04-26

**Authors:** Franco Palmisano, Luca Boeri, Matteo Fontana, Andrea Gallioli, Elisa De Lorenzis, Stefano Paolo Zanetti, Gianluca Sampogna, Matteo Giulio Spinelli, Giancarlo Albo, Fabrizio Longo, Franco Gadda, Paolo Guido Dell’Orto, Emanuele Montanari

**Affiliations:** 1 IRCCS Fondazione Ca’ Granda, Ospedale Maggiore Policlinico, Department of Urology, Milan, Italy; 2 University of Milan, Milan, Italy

## Abstract

Hospital readmission rates have been analyzed due to their contribution to increasing medical costs. Little is known about readmission rates after urological procedures. We aimed to assess the incidence and predictors of 30-day readmission after discharge in patients treated with transurethral resection of the prostate (TURP). Data from 160 consecutive patients who underwent TURP from January 2015 to December 2016 were analysed. Intra hospitalization characteristics included length of stay (LOS), catheterization time (CT) and complications. Comorbidities were scored with the Charlson Comorbidity Index (CCI). Mean (SD) age was 70.1 (8.1) yrs and mean prostate volume was 80 (20.1) ml. Mean LOS and CT were 4.9 (2.5) days and 3.3 (1.6) days, respectively. The overall 30-day readmission rate was 14.4%, but only 7 (4.4%) patients required hospitalization. The most frequent reasons for readmission were haematuria (6.8%), fever/urinary tract infections (4.3%) and acute urinary retention (3.1%). Multivariable logistic regression analysis revealed age, CCI and CT to be independent predictors of readmission. However, when analysed according to age at the time of surgery, a beneficial effect from longer CT was observed only for patients older than 75 years. These parameters should be taken in account at the time of discharge after TURP.

## Introduction

Benign prostatic hyperplasia (BPH) is a common pathologic condition that is strongly associated with ageing^1^ and is responsible for annual healthcare costs of more than $3 billion in the USA^2^. BPH commonly results in lower urinary tract symptoms (LUTS/BPH) that are known to severely affect a man’s quality of life (QoL), resulting in worsening physical and social functioning, vitality, and mental health^3^. Transurethral resection of the prostate (TURP) is the gold-standard intervention for patients with symptomatic BPH and a prostate volume of ≤ 80 mL who are refractory or cannot tolerate medical therapy^4^. Despite being considered an effective and well-tolerated surgical technique, TURP may be associated with various intra- and postoperative complications^5^, thus leading to a significant risk of post-surgical readmission.

Hospital readmission after surgery has become a topic of growing interest in the last decades and has been scrutinized for its contribution to medical costs^6,7^. Moreover, readmission rates are also considered a key metric for healthcare quality in western countries^7,8^.

Previous authors have investigated readmission rates after urological surgery. Raslan *et al*., for instance, showed 30-day unplanned readmission rate of 4.4% over 12 months in a Urology Department^9^. Similarly, Gore *et al*., reported a 31% rate of readmission within 90 days after urinary diversion while Harraz *et al*. revealed that orthotopic bladder substitution and the development of high-grade postoperative complications were significant predictors for readmission after surgery^10,11^.

However, readmission rates after common urological procedures have been scantly analysed in the current literature. To the best of our knowledge, no studies have investigated readmission rates after TURP in the real-life setting.

To this aim, we conducted a cross-sectional study assessing the incidence and predictors of 30-day readmission after discharge in a cohort of men treated with TURP for LUTS/BPH.

## Results

The initial cohort of patients included 169 men submitted to TURP but 9 patients were definitively excluded from the analysis for missing data. Between January 2015 to April 2017, 23 (14.4%) patients were readmitted to the ER within 30 days of discharge. However, readmission requiring hospitalization was reported for only 7 (4.4%) patients.

Table 1 reports the overall demographic characteristics of the cohort of patients. The mean (SD) age was 70.1 (8.0) years and mean PV was 80.1 (20.1) ml. Mean CT and LOS were 3.3 (1.6) and 4.9 (2.4) days, respectively. Patients who experienced a readmission within 30 days of discharge were older (73.5 vs. 69.4 yrs; p = 0.026) and had a higher rate of comorbidities (namely CCI ≥ 1) (78.3% vs. 51.8%; p = 0.023) than those who were not readmitted. Moreover, readmitted patients were more likely to have a POC (p = 0.015) and more frequently were under AC therapy (17.4% vs. 3.7%; p = 0.009). No differences were found with regard to BMI, educational and marital status, preoperative PSA, prostate volume, Qmax or PVR.

**Table 1:** Baseline characteristics and descriptive statistics of participants (No. = 160; mean (SD), [range]).

	Overall	Readmission	No Readmission	p-value (F)*
No. of patients (%)	160 (100)	23 (14.4)	137 (85.6)	
Age (years)	70.1 (8.0) [48–87]	73.5 (9.3) [60–87]	69.4 (7.7) [48–86]	0.026 (5.05)
BMI [kg/m^2^]	26.1 (4.1) [18.4–42.9]	25.9 (4.2) [18.4–35.0]	26.1 (4.1) [19.5–42.9]	0.83 (0.41)
CCI categorized [No. (%)]				0.023 (*X*_2_ = 5.57)
0	71 (44.4)	5 (21.7)	66 (48.2)	
≥1	89 (55.6)	18 (78.3)	71 (51.8)	
Educational Status [No. (%)]				0.33 (*X*_2_ = 0.94)
Primary/Secondary school	33 (20.6)	3 (13.0)	30 (21.9)	
High school/University	127 (79.4)	20 (87.0)	107 (78.1)	
Marital Status [No. (%)]				0.24 (*X*_2_ = 1.34)
Single	59 (36.9)	6 (26.1)	53 (38.7)	
Married	101 (63.1)	17 (73.9)	84 (61.3)	
POC [No. (%)]	55 (34.2)	13 (56.5)	42 (30.4)	0.015 (*X*_2_ = 5.97)
Time of POC (months)	7.8 (5.8) [1–32]	11.0 (8.3) [4–32]	6.7 (4.5) [1–28]	0.021 (5.67)
AC use [No. (%)]	9 (5.7)	4 (17.4)	5 (3.7)	0.009 (*X*_2_ = 6.77)
AP use [No. (%)]	53 (33.3)	10 (45.5)	43 (31.3)	0.193 (*X*_2_ = 1.69)
PSA (ng/ml)	3.1 (2.4) [0.1–9.9]	3.2 (2.6) [0.1–8.2]	3.1 (2.4) [0.1–9.9]	0.84 (0.41)
Prostate Volume (ml)	80.1 (20.1) [15–100]	77.3 (18.8) [15–100]	80.5 (23.5) [15–100]	0.76 (0.09)
Flow Max (ml/sec)	14.5 (19.1) [2.1–52.0]	18.9 (27.8) [2.1–52.0]	14.0 (18.8) [2.1–42.0]	0.48 (0.49)
PVR (ml)	126.3 (166.2) [0.0–1000]	106.1 (89.9) [0.0–500]	128.8 (173.6) [0.0–1000]	0.70 (0.15)
Surgery time (min)	106.0 (46.5) [20–180]	94.3 (37.7) [20–120]	108.0 (47.6) [30–180]	0.19 (1.71)
Hemoglobin drop (g/dl)	1.6 (1.2) [0.0–5.5]	1.5 (1.4) [0.0–5.5]	1.7 (1.1) [0.0–5.5]	0.55 (0.35)
Catheterization time (days)	3.3 (1.6) [1–14]	2.6 (0.7) [2–5]	3.4 (1.7) [1–14]	0.039 (4.33)
Length of stay (days)	4.9 (2.4) [2–19]	3.9 (0.9) [3–6]	5.1 (2.6) [2–19]	0.04 (4.26)
Complications [No. (%)]	42 (26.1)	9 (39.1)	33 (23.8)	0.124 (*X*_2_ = 2.36)
Clavien Dindo I	24 (15.0)	5 (21.7)	19 (13.8)	
Clavien DIndo II	17 (10.6)	4 (17.4)	13 (9.5)	
Clavien DIndo III	1 (0.6)	0 (0.0)	1 (0.7)	
Postoperative PSA (ng/ml)	1.1 (1.0) [0.1–5.2]	1.5 (1.2) [0.1–4.1]	0.9 (1.1) [0.1–5.2]	0.17 (0.21)

Keys: BMI = body mass index; CCI = Charlson Comorbidity Index; POC = Preoperative catheterization; AC = Anticoagulation; AP = Antiplatelet; PSA = Prostate Specific Antigen; PVR = Post void residual volume; *P value according to chi-square test or analysis of variance (ANOVA), as indicated.

With regard to perioperative outcomes, patients who experienced a readmission within 30 days had shorter catheterization times (2.6 vs. 3.4 days; p = 0.039) and shorter hospital stays (3.9 vs. 5.1 days; p = 0.04) than those who were not readmitted after TURP. The complication rate was similar between groups. Overall, complications were observed in 42 (26.1%) patients with no statistical difference between groups (p = 0.124). Postoperative hematuria and blood clot retention (Clavien I) occurred in 24 (15.0%) patients and were resolved with hydration and evacuation, respectively. Anaemia requiring blood transfusion and postoperative urinary tract infections with fever requiring antibiotics (Clavien II) were observed in 17 (10.6%) patients. Only one patient had persistent postoperative blood loss and was successfully treated with surgical haemostasis (Clavien IIIb). Patients did not differ in terms of postoperative PSA.

The most frequent reasons for ER readmission were haematuria (n = 11; 6.8%), fever/urinary tract infections (UTI) (n = 7; 4.3%) and acute urinary retention (n = 5; 3.1%). More specifically, hospitalization was necessary for 4 patients with macroscopic haematuria and 3 patients with UTI. All UTIs were symptomatic and each of these patients’ urine culture was positive for E. coli (>100,000 UFC). Patients with UTI were treated first with a wide-spectrum antibiotic (III-generation cephalosporin) and then according to antibiogram results.

Table 2 reports UVA and MVA logistic regression analysis assessing potential predictors of 30-day readmission after discharge. Univariate logistic regression analysis showed that age (OR 1.14; p < 0.001), CCI ≥ 1 (OR 5.31; p = 0.001), POC (OR 2.98; p = 0.02), CT (OR 0.28; p < 0.001), LOS (OR 0.52; p = 0.005) and AC therapy (OR 7.75; p < 0.001) were all associated with 30-day readmission after discharge. MVA analysis revealed that only age (OR 1.21; p < 0.001), CCI (OR 4.19; p = 0.04) and CT (OR 0.28; p = 0.03) were independent predictors of 30-days post-surgical readmission, after accounting for BMI, POC, AC use and length of hospital stay.

**Table 2:** Univariate and Multivariable logistic regression analysis evaluating factors associated with 30-days readmission status in the whole cohort.

	UVA		MVA	
	OR (95% CI)	p-value	OR (95% CI)	p-value
Age (years)	1.14 (1.07–1.22)	<0.001	1.21 (1.08–1.34)	<0.001
BMI [kg/m^2^]	0.98 (0.88–1.011)	0.84	1.05 (0.90–1.21)	0.53
**CCI categorized**				
0	Reference		Reference	
≥1	5.31 (1.35–8.26)	0.001	4.19 (1.21–9.53)	0.046
**Educational Status**				
Prim/Secondary school	Reference			
High school/University	0.53 (0.14–1.92)	0.33		
**Marital Status**				
Single	Reference			
Married	1.78 (0.66–4.82)	0.25		
POC	2.98 (1.21–7.34)	0.018	2.07 (0.53–6.10)	0.294
Prostate Volume (ml)	0.99 (0.98–1.01)	0.76		
Hemoglobin drop (g/dl)	0.88 (0.60–1.31)	0.55		
Catheterization time	0.28 (0.14–0.60)	0.001	0.28 (0.08–0.93)	0.038
Length of stay (days)	0.52 (0.32–0.81)	0.005	0.65 (0.29–1.44)	0.29
AC use	7.75 (1.79–6.76)	<0.001	2.33 (1.38–9.23)	0.21
Complications	2.05 (0.81–5.20)	0.129	1.34 (0.56–3.23)	0.13

Keys: BMI = body mass index; CCI = Charlson Comorbidity Index; POC = Preoperative catheterization; AC = Anticoagulation.

**Figure 1:**
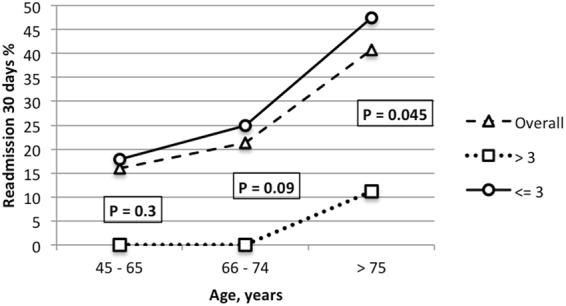
Thirty-day readmission stratified according to catheterization time (days) and age Fig. 1 depicts the catheterization times that best predict a major reduction in 30-day readmission according to age.

Figure 1 depicts the catheterization times that best predict a major reduction in 30-day readmission according to age. We found that only patients older than 75 years benefited from a catheterization time >3 days (p = 0.045).

## Discussion

The aim of our study was to assess the incidence and predictors of 30-day readmission in a cohort of patients who underwent TURP for LUTS/BPH, in the real life setting. We found an overall 30-day ER readmission rate of 14.4%, but readmission-requiring hospitalization was reported in only 7 (4.4%) patients. Patients who experienced a readmission were more likely to be older, to have higher CCI values and shorter hospital stays and catheterization times than those who were not readmitted. Moreover, readmitted patients were more likely to have a POC and more frequently on anticoagulation medication. Of clinical importance, we found a benefit from a catheterization time >3 days, but only for patients older than 75 years.

Our interest was fuelled by the lack of research in the current literature regarding the factors associated with 30-day readmission after common urological procedures. This is particularly true for TURP, which represents the gold standard treatment for symptomatic LUTS/BPH and is performed worldwide by thousands of Urologists^4^.

Previous studies examining the rate and predictors of readmission after urological surgery have focused mainly on complex procedures. Moschini *et al*. revealed a 30-day readmission rate of 12.2% in a cohort of 1090 patients treated with radical cystectomy for bladder cancer. In this study, older age and shorter LOS were associated with an increased risk of readmission after surgery^12^. Gore *et al*., instead, found a 90-day readmission rate of 31% in a large cohort of patients who underwent urinary diversion, and showed that readmitted patients had higher comorbidity counts (as defined by the CCI) than those who were not readmitted^10^. On the contrary, another study revealed early (<3 months) and late (>3 months) readmission rates of 8.6% and 11%, respectively, after radical cystectomy and urinary diversion^11^. Interestingly, the authors failed to find any associations between readmission rates and patient demographics or pathological findings, while showing that orthotopic bladder substitution and the development of postoperative high-grade complications were the only significant predictors for overall readmission^11^. Similar findings were also reported in studies assessing readmission rates in a series of patients who had undergone robot assisted radical prostatectomy (RARP)^13^. RARP patients had a low overall rate of 30-day readmission (ranging from 2.8% to 4.7% in the literature) with the main predictors of readmission being a history of advanced and/or aggressive disease and the occurrence of perioperative complications^13^.

Importantly, only a few studies have evaluated readmission rates after common urological procedures. Rambachan *et al*. analysed causes of readmission after outpatient urological surgery and showed a readmission-requiring hospitalization rate of 4.2% after TURP^8^. Moreover, they found that a history of disseminated cancer, bleeding disorder, an ASA physical status of 3 or 4, gender (male) and age were significant predictors of readmission after outpatient urological surgery^8^.

Coley *et al*., instead, reported a readmission rate of 2.9% after urological ambulatory surgery and showed that postoperative pain was the most common reason for return^14^.

Additionally, a previous study reported an overall readmission rate of 4.4% over 12 months for a Urology Department of a district general hospital, and a 30-day readmission rate of 9% following TURP^9^. The main causes of readmission were acute urinary retention (83%) and urosepsis (17%).

We performed the first study evaluating the rate and predictors of 30-day readmission after TURP for LUTS/BPH in the real life setting. Our results corroborate the previously mentioned studies on complex urological surgery as we found that age, a higher comorbidity count and a shorter LOS and CT were significantly associated with readmission after TURP. Moreover, we also showed that readmitted patients were more likely to have a POC and more frequently were assuming anticoagulation therapy, as compared to those who did not experience a readmission.

These results are of major clinical importance because they can aid physicians in the identification of patients who are at an increased risk of readmission after TURP, and may lead to the introduction of prevention strategies to reduce readmission rates in the everyday clinical practice.

The most frequent reasons for ER readmission in our cohort were haematuria, fever/urinary tract infections and acute urinary retention.

Macroscopic haematuria after TURP has been thoroughly investigated in the current literature. Normand *et al*., for example, reported a 1.6% re-hospitalization rate due to haematuria and bladder clot obstruction in a series of 624 patients submitted to TURP^15^. Our results are in line with these findings as we reported hospitalization-requiring readmission for only 4/160 (2.5%) patients for macroscopic haematuria.

Moreover, we also found that 4.3% of patients returned for fever/urinary tract infections. The type of pre- and postoperative bacterial colonization of the urine and prostate gland tissue is of major clinical importance in patients undergoing TURP. Heidler *et al*. found an heterogeneous trend in the bacterial colonization in different culture samples (pre-, postoperative urine cultures and prostate gland tissue culture) but the authors showed that the group with positive cultures was at greater risk to develop postoperative complications^16^. All patients in our cohort had negative urine cultures before surgery, but the presence of bacterial colonization in the prostate tissue, especially in patients with POC, could not be excluded.

Given that the number of patients on AC therapy has increased dramatically in recent years^17^, the management of AC therapy during TURP has been extensively debated in the current literature^18^. Previous studies have shown high rates of clot retention and late haematuria after TURP with a greater need for blood transfusions for patients on continued AC therapy^19^ compared to those not on AC therapy. Furthermore, patients on AC therapy were also rehospitalized at a greater rate^20^. We found that readmitted patients were more frequently on AC therapy and that AC therapy was associated with readmission after TURP. These results strongly support the need for greater care and attention in this specific population, which is at a higher risk for bleeding, and multimodal management of AC therapy in order to reduce post surgical complications^21^.

In line with previous studies^12^ we found that readmitted patients had a shorter LOS than those not readmitted and that a short LOS was associated with post TURP readmission. Moreover, readmitted patients also had shorter CTs than those who were not readmitted and, importantly, a shorter CT was associated with 30-day readmission in both univariable and multivariable analysis. This protective effect of longer CT (>the MOC of 3 days), on readmission was investigated, stratifying patients according to age. Importantly, only patients older than 75 years were found to benefit from a longer CT in terms of avoiding readmission.

Our study makes several important advancements with respect to previous reports on hospital readmission rates in the field of urology. First, our investigation is the only available study assessing the incidence and predictors of 30-day readmission after TURP for LUTS/BPH in the real life setting. Moreover, all of the patients in our cohort were treated at a single tertiary referral center, thus taking advantage of experienced surgeons and a high-volume setting. We also assessed incidence and predictors of 30-day readmission with the benefit of a single-center experience with a unique management strategy for TURP patients, while previous authors have investigated readmission rates in cohorts from multicenter experiences^8,14^.

Despite the potential clinical impact of our results, due to the cross sectional nature of our study, we were unable to assess the underlying mechanisms of the association between our predictors (age, LOS, CT) and readmission after TURP. However, we can speculate several likely explanations for these associations. First, older patients are typically frailer than younger ones and may be more susceptible to postoperative infections (due to their reduced immune system activity^22^). Second, older patients have a high rate of cardiovascular and metabolic disorders (as depicted by the CCI) requiring AP/AC therapy that can be responsible for the higher rate of haematuria after TURP. On the contrary, it is well known that older patients have a hypercoagulability state that may predispose them to blood clot formation and subsequent urinary retention^23^. Finally, in terms of CT, we can speculated that a prolonged catheterization time could be useful to reduce the risk of urinary retention due to the inflammatory-related oedema of the prostatic fossa after TURP.

We strongly believe that a cohort study with an independent, larger, and more diverse sample is needed to validate our results. A limitation of this study was our inability to evaluate complications and readmissions beyond the 30-day postoperative period. Moreover, precise data regarding patients’ medications other than BPH treatment and AC/AP, as well as the histological characterization of the severity of prostatic inflammation, which could all potentially impact our primary outcome, was not available. However, we believe these findings are clinically relevant due to their strong characterization in the context of the real-life setting. Our findings could help clinicians assess readmission risk during patient recovery. Particularly, we showed that AC use, patient CCI and patient age must be taken in account to assess the real benefit of longer CT, and that a strategy focused on increasing CT appears to be indicated only for patients older than 75 years. Further studies may be necessary to define what types of interventions would be most useful for reducing readmission rates after TURP.

In conclusion, we observed a 30-day ER readmission rate of 14.4% in a cohort of patients treated with TURP for LUTS/BPH. Readmitted patients were more likely to be older, have higher CCI values, and have shorter hospital stays and catheterization times than those who did not experience readmission. An increase of CT was associated with a protective effect on the risk of readmission, however this was true only for patients older than 75 years. Our findings may aid clinicians in assessing the risk of readmission after patient discharge.

## Methods

Data from 160 consecutive Caucasian – European patients who underwent bipolar TURP for LUTS/BPH at a tertiary referral center from January 2015 to April 2017 were retrospectively analysed. Demographic information, patient factors and intra-hospitalization characteristics were collected. A detailed medical history was collected for every patient. Health-significant comorbidities were scored with the Charlson Comorbidity Index (CCI; categorized 0 vs. ≥1)^24^. Measured body mass index (BMI) was considered for each patient.

Demographic information included patient age at the time of procedure, marital status and educational status. The cohort included a group of patients on anticoagulation (AC) and antiplatelet (AP) therapy. This group included patients whose AP therapy was not interrupted pre-, peri-, and/or postoperatively and patients who underwent perioperative AC bridging with low molecular weight heparin. Preoperative catheterization (POC) rate, and time of POC were also considered. Prostate Specific Antigen (PSA), prostate volume (PV), urinary maximum flow rate (Qmax) and postvoiding residual volume (PVR) were collected for every patient. A negative preoperative urine culture was required before surgery. All patients with a positive preoperative urine culture were treated in relation to the antibiogram results until the urine became sterile. Following our internal protocol, based on the local pattern of antimicrobial resistance, we used a first-generation cephalosporin as the standard preoperative prophylactic wide-spectrum antibiotic. In the case of beta-lactamase allergy, a combination of clindamycin + gentamycin was administered preoperatively.

Postoperative factors included haemoglobin drop, catheterization time (CT) and length of hospital stay (LOS). Complications were analysed according the Clavin-Dindo classification^25^. Patients received the histologic report 15 days after surgery during an office-based visit. Patients with a histologic report suggestive of incidental prostate cancer were excluded from the study (n = 4). Exclusion criteria were the presence of a known prostate or bladder cancer, a history of bladder disease or other urologic conditions likely to affect micturition after surgery, and neurogenic disorders (any type, including a positive history for overactive bladder or detrusor underactivity). Moreover, according to the current European Association of Urology Guidelines^4^ we performed urodynamic testing on patients with PVR > 300 ml and those older than 80 years. We have included in the study only cases suggestive for bladder obstruction (N = 5) and we excluded those with reported detrusor overactivity or detrusor underactivity.

Two experienced urologists, with an experience of more than 100 TURPs each, performed all of the procedures.

Patient counselling and follow-up were standardized among the cohort. All patients were instructed to return to the emergency department of the same hospital if they developed post-TURP complications. Follow-up visits for the evaluation of possible complications were scheduled 15 and 60 days after surgery as per standard clinical protocol. An assessment of PSA, urinary flow rate and PVR were scheduled 2-months after surgery.

The primary outcome was defined as admission to the emergency room (ER) of the same or a different hospital for a postoperative complication related to primary surgery within 30 days of discharge. Readmission rates were investigated either during the scheduled follow-up visit or with phone calls to patients who were lost at follow up 2 months after surgery.

The primary endpoint of the study was to assess the rate of readmission within 30 days in our cohort of patients treated with TURP for LUTS/BPH. We also evaluated potential factors associated with readmission after TURP.

Data collection was carried out following the principles outlined in the Declaration of Helsinki; after approval of the IRCCS Fondazione Ca’ Granda – Ospedale Maggiore Policlinico Ethical Committee, all patients signed an informed consent agreeing to supply their own anonymous data for this and future studies.

Data are presented as means (SD; ranges). The statistical significance of differences in means and proportions was tested with the one-way analysis of variance (ANOVA) and Pearson chi-square test, respectively. A 95% confidence interval (95% CI) was estimated for the association of categorical parameters. Exploratory analyses were initially applied to all variables; variables were retained for analysis when deemed clinically significant to the results. Descriptive statistics were used to assess potential differences in terms of clinical parameters and perioperative characteristics according to the readmission within 30 days status.

The number of days of catheterization after surgery was dichotomized according to the most informative cutoff (MOC) predicting 30-day readmission (3 days). This value was obtained by applying the X^2^ test for every possible cutoff value and choosing the lowest P value. Finally, patients were stratified into age categories and the most informative CT was applied to each category, testing differences in 30-day readmission after discharge. Univariate (UVA) and multivariable (MVA) logistic regression analyses were performed to assess the relationship between preoperative and perioperative characteristics and the probability of 30-day readmission. Statistical analyses were performed using the R statistical package system version 3.0.2 (R Foundation for Statistical Computing, Vienna, Austria) and SPSS statistical software, v 13.0 (IBM Cor., Armonk, NY, USA). All tests were two sided, with a significance level set at 0.05.

### Data Availability Statement

All relevant data are within the paper and its Supporting Information files.
